# Economy in the Generation and Use of Steam

**Published:** 1915-01-23

**Authors:** 


### ECONOMY IN THE GENERATION AND USE OF STEAM.*

### Waste of Heat in the Boiler.

Among sources of waste of heat in the boiler, one arises from the salts that are contained in a good deal of the water that is available for converting into steam, and also from the oil, which has a trick of working back into the boiler, whenever the exhaust steam is condensed. In hospitals where steam is employed for heating the water and a steam engine is employed to furnish power, it is economical to use the steam which has already done its work in the steam engine (the "exhaust steam") for the purpose. The major portion of the heat required to convert water into steam is known as the "latent" heat of the steam.

When steam is used in calorifiers to heat water for various purposes, it gives up this latent heat, and hence the arrangement is a very economical one; but when the steam carries with it the vapour of oil, some of the latter may be deposited upon the tubes in the calorifier, and some of it passes on in the water formed by condensation and is carried back into the boiler. The oil very often deposits upon the crowns of the furnaces in the case of Lancashire boilers, and upon the insides of the tubes through which the water circulates in the case of water-tube boilers. The salts which are carried in solution by much of the water available for raising steam are also deposited upon various parts of the boiler, upon the hot surfaces for preference; and there is a particularly troublesome deposit that is formed by the union of the oil with the salts from the water. The deposit of oil, and the "scale," as the deposit of salts or of salts and oil is called, set up a resistance to the passage of heat from the hot gases liberated by the combustion of the coal in the furnace, to the water or steam to which the heat should be delivered.

This necessitates a larger consumption of coal than would otherwise be necessary; and in addition, in the case of Lancashire boilers, it sometimes leads to the collapsing of the furnace crowns; also, in water-tube boilers, to the burning of some of the tubes. The hot gases which rise from the fuel in the furnace are at a temperature in the neighbourhood of 2,000° Fahr. and upwards; while the water on the other side of the metal heating surface is only in the neighbourhood of 200° Fahr.; therefore, if anything prevents the heat passing freely from the hot gases to the water, the plate or pipe against which the hot gases impinge has its temperature very dangerously raised, with the result that oxidation takes place, and in some cases burning out, or collapse.

The remedy for both of these troubles is to eliminate the oil from the steam and the salts from the water.

The oil separator works upon the principle that the temperature at which oil is able to maintain the vaporous condition is higher than that at which steam condenses, other conditions being the same. The steam in which the vapour of oil is present in flowing through the oil separator is checked by what are called "baffles"; the oil is condensed upon the baffles, and runs down into receptacles provided for it. Oil separators are of very various forms; but in all of them the steam from which the oil is to be abstracted is given a more or less tortuous path. Plates of various forms are interposed in its path in such a manner as to trap the oil by causing it to condense upon their surfaces. The success of oil separators depends very largely upon having a sufficiently large baffling surface, and on giving the steam a sufficiently long path to trap all the oil vapour. Care must be taken not to throttle the steam in its passage through the oil separator. This is ensured by making the area of the passages through which the steam passes sufficiently large.

The salts that are present in water with which boilers are fed are abstracted by apparatus known as "water softeners." To abstract salts in the water softener the water is heated, so that what happens in the boiler itself happens before the water enters the boiler; and the salts are deposited in the water softener, the water passing on freed.

Another class of salts which make the water hard is neutralised by the aid of lime and carbonate of soda, the resultant salts being removed by filtration and by deposition in the water softener. Great care is necessary that the quantities of these salts that are added to the water to be softened are exactly in proportion to the salts to be removed.

* Previous articles on aspects of this subject appeared last year on May 30, June 13, and July 25.

